# Targeted delivery of extracellular vesicles: the mechanisms, techniques and therapeutic applications

**DOI:** 10.1186/s43556-024-00230-x

**Published:** 2024-11-21

**Authors:** Shuang Zhao, Yunfeng Di, Huilan Fan, Chengyan Xu, Haijing Li, Yong Wang, Wei Wang, Chun Li, Jingyu Wang

**Affiliations:** 1 College of Traditional Chinese Medicine, Beijing University of Chinese Medicine, Beijing, 100029 China; 2 Dongzhimen Hospital, Beijing University of Chinese Medicine, Beijing, 100029 China; 3 Key Laboratory of Traditional Chinese Medicine Syndrome and Formula, Ministry of Education, Beijing, 100029 China; 4 State Key Laboratory of Traditional Chinese Medicine Syndrome, Guangzhou University of Chinese Medicine, Guangzhou, 510006 China; 5 Modern Research Center for Traditional Chinese Medicine, Beijing University of Chinese Medicine, Beijing, 100029 China

**Keywords:** Extracellular vesicles, Exosome, Targeted delivery, Engineered extracellular vesicles, Targeting mechanism, EV engineering technique, Clinical translation

## Abstract

Extracellular vesicles (EVs) are cell-derived vesicles with a phospholipid bilayer measuring 50–150 nm in diameter with demonstrated therapeutic potentials. Limitations such as the natural biodistribution (mainly concentrated in the liver and spleen) and short plasma half-life of EVs present significant challenges to their clinical translation. In recent years, growing research indicated that engineered EVs with enhanced targeting to lesion sites have markedly promoted therapeutic efficacy. However, there is a dearth of systematic knowledge on the recent advances in engineering EVs for targeted delivery. Herein, we provide an overview of the targeting mechanisms, engineering techniques, and clinical translations of natural and engineered EVs in therapeutic applications. Enrichment of EVs at lesion sites may be achieved through the recognition of tissue markers, pathological changes, and the circumvention of mononuclear phagocyte system (MPS). Alternatively, external stimuli, including magnetic fields and ultrasound, may also be employed. EV engineering techniques that fulfill targeting functions includes genetic engineering, membrane fusion, chemical modification and physical modification. A comparative statistical analysis was conducted to elucidate the discrepancies between the diverse techniques on size, morphology, stability, targeting and therapeutic efficacy in vitro and in vivo. Additionally, a summary of the registered clinical trials utilizing EVs from 2010 to 2023 has been provided, with a full discussion on the perspectives. This review provides a comprehensive overview of the mechanisms and techniques associated with targeted delivery of EVs in therapeutic applications to advocate further explorations of engineered EVs and accelerate their clinical applications.

**Supplementary Information:**

The online version contains supplementary material available at 10.1186/s43556-024-00230-x.

## Introduction

**Fig. 1:**
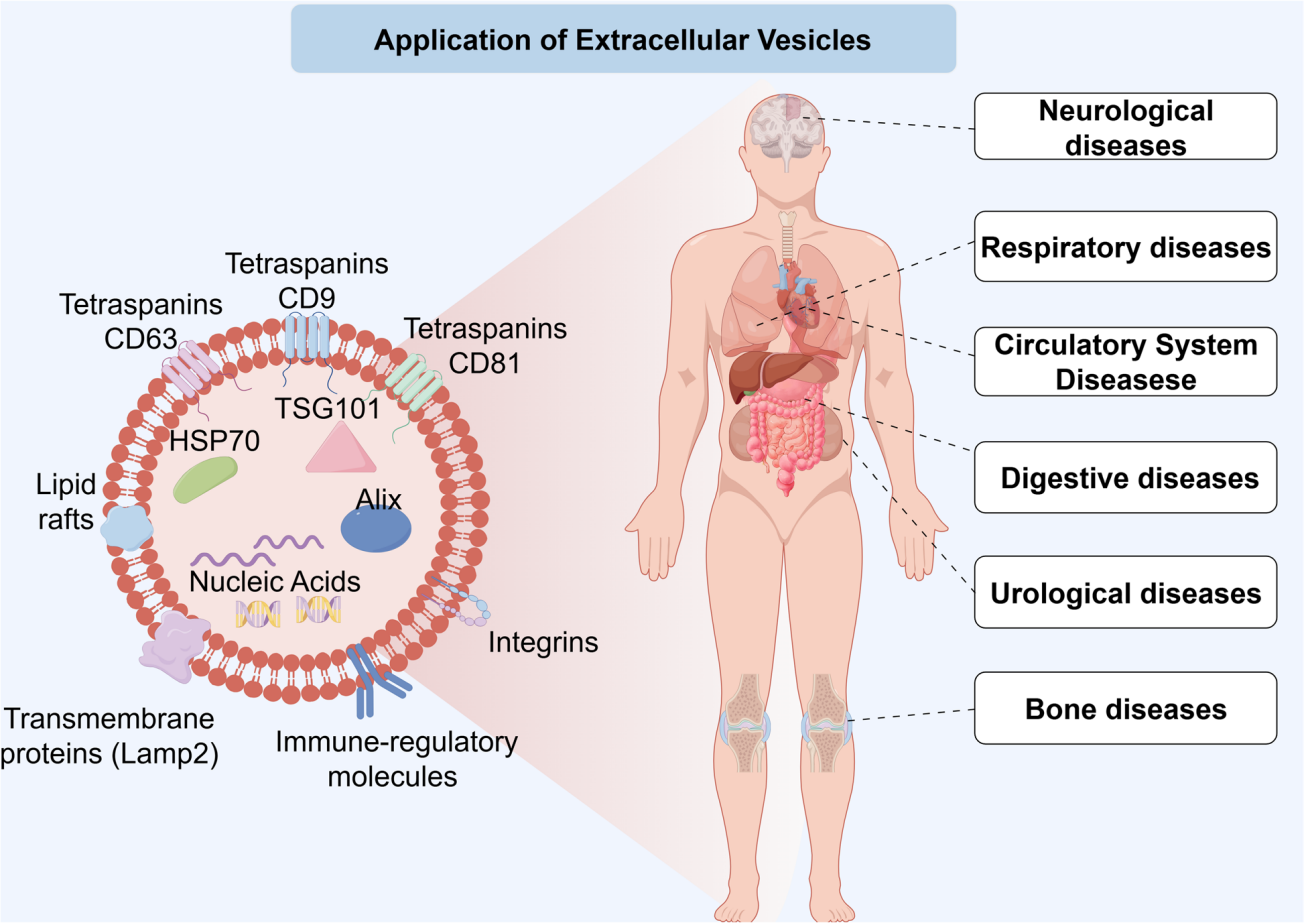
Overview of engineered EVs applications. Engineered EVs can be used for treating variety of diseases. Elements were obtained from Figdraw

Extracellular vesicles (EVs) are cell-derived vesicles with a 50–150 nm diameter, which carry proteins, nucleic acids, lipids and other biomolecules. Endogenous EVs play an important role in the initiation and progression of disease [1–5]. Breast cancer cell-derived EVs have been shown to promote proliferation, migration, epithelial-mesenchymal transition (EMT) and angiogenesis by inducing tumor-associated macrophage polarization [6]. While exogenous EVs have excellent therapeutic potential in the treatment of a variety of diseases (Fig. 1) [7–10]. For the treatment of a plethora of diseases, a multitude of nanoparticles and nanoscale drug delivery vehicles have been developed based on natural and artificial polymers, for example, dextran nanoparticles [11]. However, in comparison to EVs, nanoparticles indicated poor capacity to carry multiple active ingredients in consideration of inner space, which limits their utility for drug delivery. The safety and stability issue of nanoparticles are also a sustaining concern associated with their artificial origin. EVs are of cellular origin with a vesicular structure, exhibit good biocompatibility and delivery ability, and thus display great potential for therapeutic applications with further developments [12–15].

EVs indicated significant advantages in the treatment of neurological disorders by increasing the capability of passing the blood-brain barrier (BBB). The BBB represents a protective physiological obstacle in the treatment of neurological disorders, as exogenous components are limited to traversing this barrier. Natural EVs possess favorable biocompatibility and are capable of crossing the BBB, thereby offering a promising avenue for the treatment of neurological diseases [16]. It has also been demonstrated that targeted modification serves to augment the therapeutic effects of EVs derived from induced neural stem cells (iNSCs) on inflammatory response, apoptosis and neural repair after spinal cord injury [17]. Moreover, EVs represent a novel therapeutic tool with significant potential for the treatment of cardiovascular disease [18]. Their mechanisms of action encompass a range of processes, including promotion of angiogenesis, inhibition of myocardial fibrosis, reduction of apoptosis, and modulation of immune responses. EVs from hypoxia-preconditioned mesenchymal stem cells have been demonstrated to promote angiogenesis after myocardial infarction [19]. EVs have been observed therapeutic effects via various administration routes. The intravenous administration of EVs, the most often used route, has been demonstrated to facilitate neurological recovery, attenuate inflammation and promote cerebral remodeling following distal middle cerebral artery occlusion in rats [20]. Local injection, for example, intracardiac injection of stem cell-derived EVs has been shown to promote angiogenesis after myocardial infarction [21]. Inhalable stem cell EVs have been demonstrated to facilitate cardiac repair following myocardial infarction [22]. Furthermore, studies have employed hydrogels in conjunction with EVs to facilitate dermal administration of pharmaceuticals to enhance diabetic wound healing [23].

Currently, the clinical translation of EVs is still facing significant challenges. Firstly, most EVs used in clinical trials are derived from conditional cell culture medium and there is a lack of mature technology for large-scale production, which results in low yields and a high cost of EVs [24]. Secondly, the targeting of natural EVs to specific organs is ineffective. Following intravenous injection, natural EVs are primarily accumulated in the liver and spleen, with a half-life in the blood of approximately 10–30 min for most types [25–28]. Finally, the in vivo tracking of EVs remains a challenge, and the lack of clarity on the pharmacokinetics makes it hard to eliminate concerns about potential adverse effects. It is therefore of great urgency to develop engineering technologies for EVs to improve their accumulation at the lesion site [29, 30].

This review presents an overview of the prevailing engineering methods and challenges associated with the development of targeted delivery of EVs. Additionally, further statistical analysis are provided concerning the impact of disparate modification techniques and molecules on targeting and treatment efficacy in the context of ischemic heart disease. Moreover, an overview of the registered clinical trials related to EVs is provided, accompanied by a discussion of their findings on safety. Finally, the article concludes with an overview of potential future developments of applying EVs in targeted delivery from our perspectives.

## Targeting mechanisms in EV engineering

**Fig. 2:**
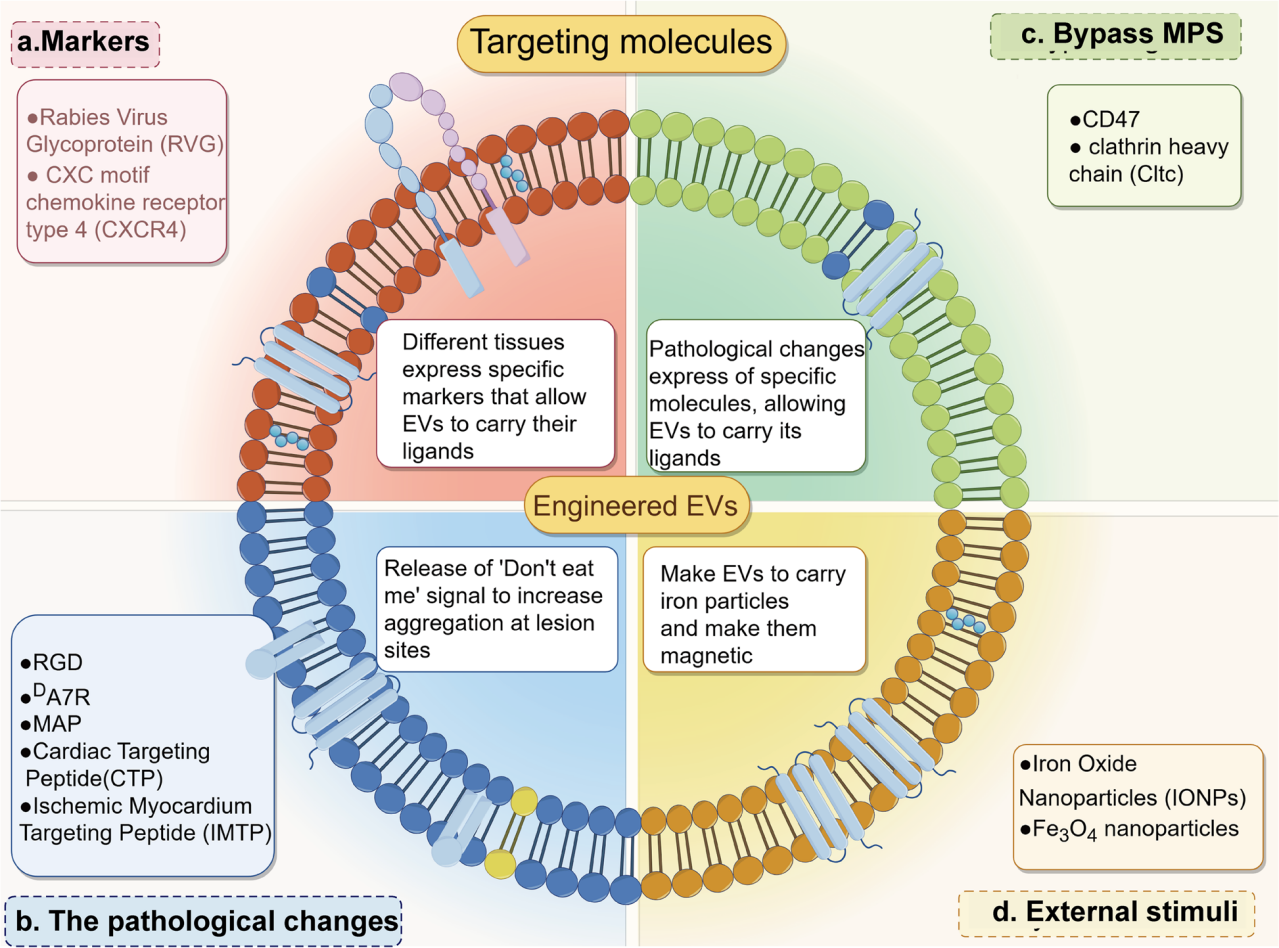
The classification for targeting molecules. Targeting molecules are classified according to the principles on which they are based, as follows: marker (**a**), pathological changes (**b**), bypassing MPS (**c**) and external stimuli (**d**). Elements were obtained from Figdraw

Tissue-specific binding or bypassing phagocytosis capacity could facilitate the accumulation of EVs on lesion sites. Tissue-specific binding of EVs could be achieved by modifications of EVs according to tissue markers, pathological changes or physical interactions. In addition, ligands could be added to the EV membrane to decrease mononuclear phagocyte system (MPS) phagocytosis in the liver and spleen, the primary accumulation sites of EVs following oral or intravenous administration, and thereby extend the circulation time [31]. The targeting molecules that have been exploited to deliver EVs or cargo-bearing EVs as potential new therapies in recent publications are reviewed in Fig. 2.

### Tissue markers as targets

Markers expressed in the membranes of specific cell types, such as neurons and endothelial cells, have been employed as binding objects in engineered EVs.

Acetylcholine receptors (AChR) are mainly synthesized and released by neuronal cells located in the central regions of the brain and the kidney [32, 33]. Rabies virus glycoprotein (RVG) peptides have been developed for the selective binding of AChR [34]. In a model of traumatic brain injury, the accumulation of RVG modified EVs in the brain was increased approximately 2.8-fold, as that in the mouse brain of the cerebral ischemia model, Machado-Joseph model, and chronic unpredictable stress model [35–38]. There have also been studies using RVG-EVs to treat renal fibrosis, which resulted in a 1.38-fold increase in EV accumulation in the kidney, thereby improving renal tubulointerstitial fibrosis [39, 40].

Stromal cell-derived factor-1 (SDF-1), also known as chemokine C-X-C motif ligand 12 (CXCL12), is mainly expressed in bone marrow stromal cells and bone marrow endothelial cells and can be recognized by the CXC motif chemokine receptor type 4 (CXCR4) [41]. CXCR4 overexpressing EVs (CXCR4-EVs) derived from E. coli or 3T3 cells showed high homing potency to the bone marrow, thus providing a promising avenue for the treatment of bone related diseases. CXCR4-EVs have been used for the treatment of osteoporosis with improved bone retention and efficacy [42–44]. SDF-1 is also highly expressed in inflammatory tissue. CXCR4-EVs have been employed in treating acute myocardial infarction, cerebral ischemia-reperfusion injury, ulcerative colitis, periodontitis, and glioblastomas to target inflammatory lesions and improve treatment efficacy [45–55].

### Pathological changes as targets

The pathological alterations observed at the site of a lesion represent a significant source of binding sites for the targeted delivery of EVs. It is frequently observed that pathological tissues exhibit expression changes of specific proteins, including cell adhesion molecules, glycoproteins, growth factor receptors, etc. In response to these changes, peptides and nucleic acids are designed to enable targeted delivery of EVs to lesion sites.

The expression of integrin αvβ3, a major component of the integrin family of transmembrane adhesion receptors, is elevated at neovascular sites and in tumors (e.g., osteosarcoma) [56]. Arginine-glycine-aspartic acid (RGD) peptide consists of three amino acids with a linear or cyclic structure and binds specifically to αvβ3. Consequently, engineering EVs using RGD improves the targeting of EVs. The cyclic structure of RGD (cyclo(RGDyK)) is more stable and has a higher binding affinity to the integrin receptor [57]. Cyclo(RGDyK) peptide-engineered EVs exhibited superior aggregation to the lesion area, with an 11-fold increase [58]. Other RGD-based peptides, including c(RGD-^D^Phe-C) (an RGD peptide with an amino acid Phe in an unnatural D-conformation), iRGD (an RGD peptide with a cryptic CendR motif and cell-penetrating activity) [59], and RGD-4 C (an RGD peptide with a lactadherin C1C2 domain) [60], have been employed for EV modifications to achieve specific targeting to tumors. The modification of EVs to enable selective targeting represents a further potential avenue for their use as delivery vehicles, with the capacity to transport a range of payloads, including small molecules, nucleic acids and proteins [61]. Bone marrow mesenchymal stem cell (BMSC)-derived EVs modified with an integrin α₅-targeting peptide were found to enhance the efficacy of therapeutic regimens targeting pancreatic cancer by promoting the reprogramming of cancer-associated fibroblasts (CAFs) while inhibiting the pro-carcinogenic effects [62]. A study utilizing iRGD peptide-modified EVs for targeted delivery of doxorubicin (DOX) demonstrated that the modified DOX@EVs markedly inhibited tumor growth while exhibiting increased biosafety and reduced adverse effects [63].

CD44 is a cell surface adhesion molecule overexpressed on cancer stem cells [64], thus applied in surface modification of Doxorubicin (DOX) loaded EVs for cancer therapy. Meanwhile, the pH-responsive 3-(diethylamino)propylamine (DEAP) was modified on functional EV for the local release of DOX in response to the acid tumor microenvironment [65–67]. CD44 is also overexpressed in the context of fibrosis while at a relatively low expression level in the normal liver. CD44 could bind specifically to hyaluronic acid (HA), a biocompatible carbohydrate polymer that has been approved by the U.S. Food and Drug Administration (FDA) for use in drug delivery systems. Therefore, HA-engineered EVs facilitate interaction with CD44-specific ligand receptors and targeted delivery of coniferin A, the anti-hepatic fibrosis compound, to fibrotic tissue. The anti-hepatic fibrosis effect of coniferin A was thus enhanced through increased EV accumulation at the lesion site [68].

Glycoprotein P-selectin is a biomarker of ischemic acute kidney injury (AKI) in endothelial cells [69]. Consequently, a P-selectin binding peptide (PBP) was added to the membrane of EVs (PBP-EVs) through hydrophobic interactions. The results demonstrated that PBP-EVs exhibited a selective targeting tendency to damaged kidneys as visualized using molecular imaging [70]. ^D^A7R (^D^R^D^P^D^P^D^L^D^W^D^T^D^A) peptide has a high binding affinity to vascular endothelial growth factor receptor 2 (VEGFR2) and neuropilin-1 (NRP-1) in damaged blood vessels. EVs with ^D^A7R peptides on the surface showed a 1.57-fold increase in the ischemic cerebral regions after intravenous injection, which improved the differentiation of NSCs into neurons after stroke [58, 71–79].

Some of the targeted peptides are capable of penetrating cellular membranes, hence their designation as cell-penetrating peptides (CPPs), also known as protein transduction structural domains (PTDs). These peptides exhibit cell-penetrating properties as a consequence of the presence of basic amino acids [80]. Furthermore, engineered arginine-rich CPPs on EV membranes leads to activation of the giant cytosolic drinking pathway, and the number of arginine residues in the peptide sequence influences the efficiency of cellular EV uptake [81]. CPPs include those with targeting properties, such as iRGD, MAP, etc., and those without targeting properties, such as Trans-activator of transcription (TAT) peptide [82], which only have membrane-penetrating properties, could be integrated with other targeting peptides without membrane-penetrating properties to reduce the effect of receptor saturation [83].

Metal abstraction peptide (MAP) is a cell-penetrating peptide targeting matrix metalloproteinases (MMP) [84]. MMP-9 levels are elevated in the brain tissue of patients with ischemic and hemorrhagic stroke. Engineered EVs by MAP via hydrophobic interactions (MAP-EV) were developed for ameliorating ischemic stroke. After 12 h of intravenous injection, MAP-EVs were significantly enriched in the ischemic zone than in the control group, and this difference persisted up to 48 h [85, 86]. The dual tumor-penetrating peptides iRGD and tLyp1-modified EVs demonstrated a markedly enhanced uptake of doxorubicin (Dox) by MCF-7 and MDA-MB-231, the breast cancer cell lines, compared to single iRGD or tLyp1-engineered EVs or EVs alone [87].

However, the application of CPP presents a number of challenges, the first of which is toxicity. The toxicity of CPP is primarily contingent upon the peptide concentration, the specific cargo molecule, and the employed coupling strategy [88]. Amphiphilic CPPs tend to exhibit heightened toxicity relative to their cationic counterparts. The second challenge is stability [89]. Extracellular and intracellular CPPs could be degraded by proteases and lose the capacity for delivery. The potential strategy to improve metabolic stability is the end-to-end cyclisation of CPPs [90]. Thirdly, some CPPs have non-specific tissue and cell permeability, which limits the application in vivo [91]. Regarding this issue, CPPs are integrated with peptides that are designed with tissue-targeting properties for engineering EVs. TAT peptide is able to penetrate the plasma membrane as well as the nuclear envelope of most living cells, while Angiopep-2 is an active target peptide with a high affinity for LRP1 in brain. Combining Ang and TAT, the dual peptide-engineered EVs not only exploited the efficient TAT-mediated cell membrane penetration and Ang-mediated targeting ability against gliomas, but also overcame Ang receptor saturation [92]. The targeting ability of RGD peptides and the cell-penetrating ability of Angiopep-2 peptides engineered EVs have shown that dual-engineered EVs significantly enhance transcellular permeability across the BBB in vivo. The engineered EVs presented targeting to ischemic blood vessels after intravenous administration, and also achieved rapid accumulation in the ischemic lesion area [93].

Besides known markers and pathological changes, Moreover, the targeting capacity could be achieved through peptide screening. The utilization of phage display technology enables the discovery of hitherto targeting peptides before identifying the unknown target peptides. This is achieved by employing target proteins (or target, cells or tissues) as bait, incubating them with a phage peptide library, and subsequently screening out the peptide sequences that interact with the target after rounds of binding, elution and amplification [94]. The resulting peptides have proved their effectiveness for EVs engineering, such as iRGD (internalizing-RGD) [72], CTP (cardiac targeting peptide, peptide sequences: APWHLSSQYSRT) [92, 93, 95, 96], IMTP (ischemic myocardium-targeting peptide, peptide sequences: CSTSMLKAC) [18, 97–104], HHP (heart homing peptide, peptide sequences: CRPPR) [105], CMP (cardiomyocyte specific peptide, peptide sequence: WLSEAGPVVTVRALRGTGSW) [106]. The CTP modification resulted in an approximate 16% improvement in the uptake of EVs by cardiomyocytes, while no significant enhancement was observed in the uptake by other cells. Furthermore, CTP modification resulted in a 2.44-fold increase in the accumulation of EVs in the heart at 24 h post-intravenous injection [96]. Compared to EVs, CTP-engineered EVs have presented a more therapeutics on cardiac repair following viaral myocarditis by modulating the immune microenvironment, such as regulating macrophage polarization [107]. Concurrently, IMTP-EV has been observed to stimulate cell proliferation and angiogenesis by enhancing targeting to the infarcted heart, while simultaneously reducing fibrosis and scar formation [104]. HHP engineered EV localized in mouse cardiac tissue via selective binding to cysteine-rich proteoglycans. Consequently, HHP modification resulted in an increase in the accumulation of EVs in the heart [108].

In addition to proteins, nucleic acids can also be used for targeted modifications such as aptamers. Aptamers are oligonucleotides that are capable of binding to multiple ligands with high affinity and specificity, representing a novel class of EVs modification molecules [109]. AS1411 is a stable G-quadruplex-forming DNA sequence that binds to nuciferine (NCL) with high affinity and specificity, acting as a target protein [110]. NCL is overexpressed in the cytoplasm and cell membrane of a variety of tumors. To increase the EV binding to NCL, the 5-terminal carboxylic acid group of the AS1411 aptamer was converted to an amine-responsive NHS ester by EDC/NHS amide-coupled chemistry, which was then coupled to an amine group on the surface of the EVs. This resulted in an increased accumulation of AS1411 adaptor-modified EVs at tumor sites [111, 112].

### Bypassing MPS for elongated circulation time

EVs in circulation were eliminated quickly through phagocytosis by the mononuclear phagocyte system (MPS) in the liver or spleen. Reduced phagocytosis and prolonged cycling time of EVs could be achieved by adding protein or peptides, such as CD47, clathrin heavy chain 1(Cltc) and so on, by releasing the ‘don’t eat me’ signal.

CD47 is an integrin-associated glycoprotein and could be recognized by signal regulatory protein α (SIRPα) of macrophages, reducing phagocytosis in the liver or spleen [113–115]. The membrane expression of CD47 of EVs has prevented their clearance by circulating monocytes [116, 117]. CD47 overexpressed EVs (CD47-EVs) prolong the circulation time of EVs from 30 to 120 min post-injection [118]. In addition, CD47 is highly expressed on cardiac resident macrophages (cMacs), so modification of EVs with cMacs membrane can also increase the immune evasion capacity, allowing for a longer circulation time from 12 to 24 h [119]. Cltc (clathrin heavy chain) has also been shown to play an important role in mediating exocytosis in the liver and spleen. By knocking down Cltc, the endocytosis of MPS into exocytosis in the spleen and liver can be significantly and specifically blocked, thus facilitating the efficient arrival of EVs to other tissues such as the heart. It has been shown in DOX-induced cardiomyopathy (DIC) model that Cltc@CTP-EVs led to a reduction in the internalization of the MPS, resulting in a reduction of 24.64% in the liver and 36.74% in the spleen [120, 121]. In addition to direct modification of EVs to reduce phagocytosis, it is also possible to prolong circulation time by preemptively blocking phagocytic receptors. Commonly used receptors include Phosphatidylserine (PS), Galactose lectin-5 (Gal-5), and Scavenger Receptor Class A family (SR-A). PS exposed to the surface of EVs and recognized by macrophages may also affect macrophage clearance. Previous studies have found that pretreatment with negatively charged PS liposomes reduced the fluorescence intensity of EVs from 40 to 26% in liver [122]. Gal-5 is a predominantly cytoplasmic fraction of the lectin superfamily, and it has been found that Gal-5 binds to the surface of the EVs of rat reticulocytes. The addition of Gal-5 has reduced vesicle uptake by macrophages [123]. SR-A recognizes a variety of negatively charged ligands on EVs. A using dextran sulfate significantly reduced EVs clearance by mouse liver in vivo, while tumor uptake increased approximately 3-fold over 24 h [124].

### External stimuli enabled targeted delivery of EVs

Tissue accumulation of EVs can be achieved not only by the recognition of specific molecules, termed as “intrinsic- stimuli”, but also by external stimuli such as magnetism, ultrasound and light. The utilization of magnetism is facilitated by the incorporation of Iron Oxide Nanoparticles (IONPs), which can be enriched at the lesion site in the presence of an external magnetic field [125]. The IONPs are biocompatible and easy to be incorporated into the membrane and inner space of EVs. Using membrane filters and a mini-extruder, IONPs were encapsulated into MSC derived EV mimetics (magnetic EM, M-EM). EMs are EV-like vesicles that are produced through direct artificial manipulation with similar size, morphology, marker (such as tetraspanins CD9, CD63 and CD81) and delivery capacity [126]. The M-EM improved the targeting to ischemic foci by 2.9-fold and a significant enhancement in the treatment efficacy in ischemic stroke [127, 128]. In addition, IONP-engineered EVs have good potential in cancer therapy by enhancing the targeting and sacrifice of tumors. The ionization of IONPs at the acidic tumor microenvironment would release metal ions Fe²⁺ that undergo a Fenton or Fenton-like reaction to convert excessive H₂O₂ to •OH. IONPs@EMs have shown enhanced uptake by ovarian cancer cells in a magnetically guided manner, exhibiting superior tumor targeting and suppression without significant side effects [129, 130].

Ultrasound-targeted microbubble disruption (UTMD) technology represents a promising approach for targeted EV delivery [131]. By emitting ultrasound waves of varying intensities at a specific site, microbubbles in the blood are ruptured, resulting in the release of energy that can increase the permeability of cell membranes and blood vessels. This facilitates the delivery of the therapeutic agent to the desired site, thereby enabling targeted therapy [132]. Utilizing this principle, targeted delivery of EVs was enhanced to heart, liver, spleen and adipose tissue when the EVs were irradiated [133]. The enhanced accumulation of EVs in the heart were achieved after an injection of EVs in six minutes after UTMD [134]. The focused ultrasound and microbubble technology was utilized to achieve brain-targeted EV delivery by temporarily open the BBB. This approach enables EVs to cross the BBB more efficiently and reach the brain, thereby facilitating the prevention of amyloid beta plaques and providing neuroprotection [135].

## Techniques in EV modification

**Fig. 3:**
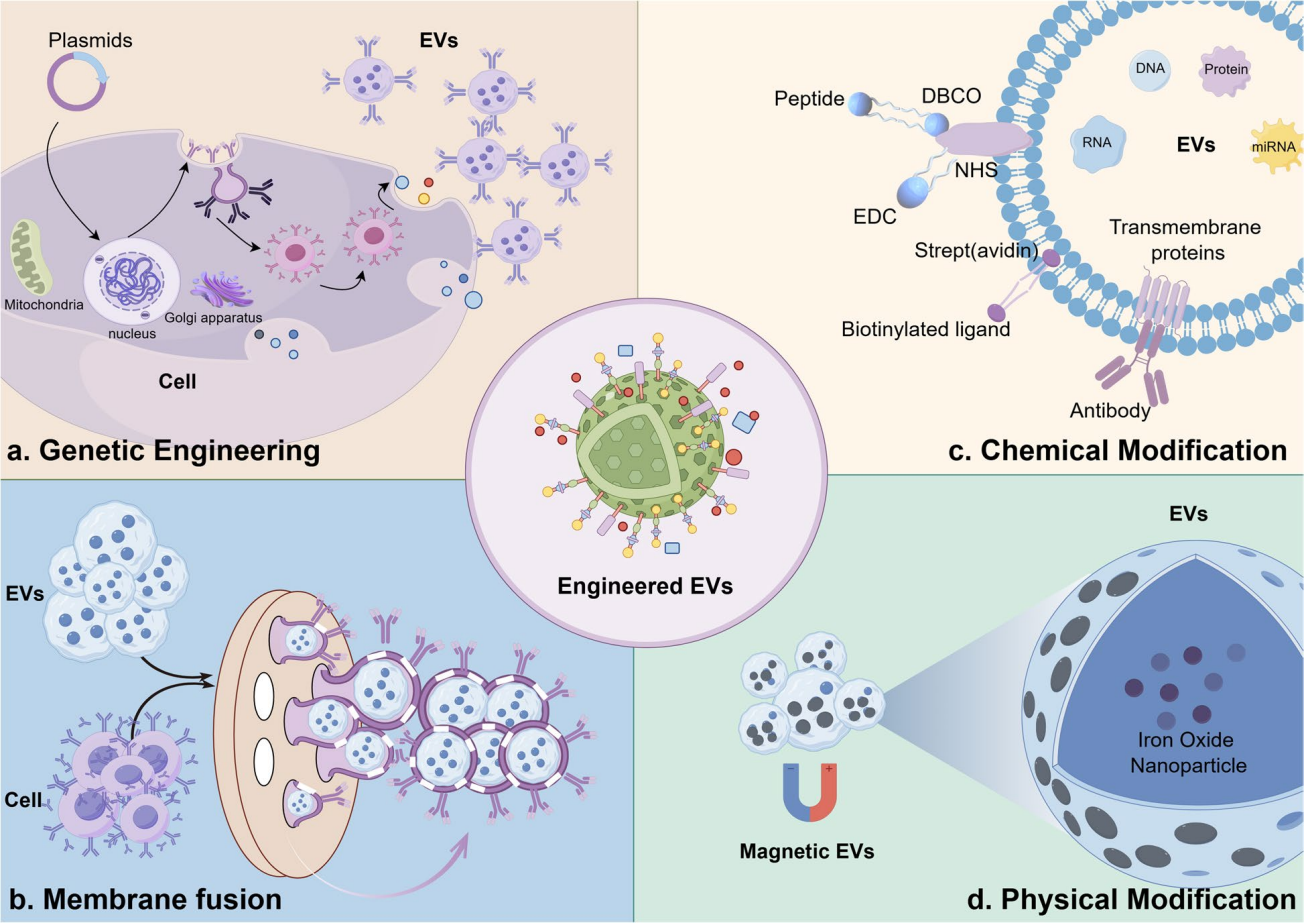
The classification for engineering techniques. Commonly used engineering methods of EVs include genetic engineering (**a**), membrane fusion (**b**), chemical modification (**c**) and magnetism (**d**). Elements were obtained from Figdraw

In modifying EVs with various molecules, the most commonly used methods include genetic engineering, membrane fusion chemical modifications, and physical modification. These techniques helped to prolong circulation time and increase targeting by binding peptides or proteins on EVs membranes (Fig. 3).

### Genetic engineering

Genetic engineering technology constructed plasmids for fusion proteins comprising EV membrane surface proteins, such as CD63 and Lamp2b (lysosomal associated membrane protein 2b), in conjunction with specific targeting peptides, including IMTP, CTP, c(RGDyK), RVG and TAT, and ligands such as CXCR4, Angiopep-2(Ang-II) and CD47. Such plasmids are transfected to the parental cells, thereby integrating the targeting capacity to secreted EVs [104, 136, 137].

By genetic engineering, the newly constructed parental cells express fusion proteins stably. However, this method was limited by time-consuming operations with complex procedures, and varied transfection efficiency depending on the parent cell types [138]. In addition, targeting peptides on the membrane surface of engineered EVs may be degraded during EV maturation and secretion. A potential option is adding glycosylation sequences to the plasmid of the fusion protein to prevent degradation [139, 140]. In addition, the biological functions and stability of fusion proteins on EVs require careful experimental examinations in vitro and in vivo before clinical applications in the face of ethical and safety challenges.

### Membrane fusion

Membrane fusion is to fuse the membrane of therapeutic EVs with another cell membrane containing the targeting molecules through the application of extrusion or repeated freeze-thawing. Membrane of platelet and monocyte are most widely used as source of membranes that contain targeting molecules since their natural response to recruitment of the inflammatory tissue [141–147].

Platelets were used to modify MSC derived EVs since their natural targeting properties to atherosclerotic plaques. Platelet membranes modified EVs (P-EVs) aggregated at injured endothelial cells in vitro, and atherosclerotic plaques in the aorta or heart in vivo, indicating improved atherosclerotic treatment [148–150].

The advantage of membrane fusion is that it is easy to follow and requires only physical manipulation. However, there are also shortcomings. It is difficult to ascertain whether membrane proteins are affected during extrusion, and whether active substances such as miRNA are affected. The membrane fusion is easy to follow with only physical manipulations. However, further research is required to elucidate the optimal methods for maintaining the functionality of cell membranes during the modification of EVs. Given that cell membranes constitute a vital component of living organisms, they are susceptible to a multitude of environmental factors during the modification process, including temperature, the specific reagents employed, and substrate concentration. It is difficult to ascertain the distribution, potion and biological activity of membrane proteins in fusion. The loss of therapeutic contents, such as miRNA, in the process of membrane disrupture required further optimizations on the physical manipulations. The development of effective techniques for verifying the success of membrane fusion and quantifying the proportion of each membrane in the synthesized new membrane represents a crucial avenue for further investigation. Moreover, the supply and regulation of mammalian platelets or monocytes as membrane sources in EV engineering also raise concerns in clinical applications [151].

### Chemical modification

Chemical modification is connecting the targeting peptide or ligands on the surface of EVs membranes to enhance their targeting properties by click chemistry reactions, copper-free click chemistry reactions, carbodiimide chemistry, antigen and antibody interactions, and biotin-avidin binding.

Click chemistry reactions are based on copper-catalyzed azide-alkyne cyclisation to bind the target peptide or ligand to the EV surface [152]. Click chemistry reaction is used to bind glioma-specific ligand RGERPPR peptide (RGE) on the surface of EVs, improving the targeting of EVs to gliomas [153]. Canonical click chemistry is catalyzed by copper ions. However, the copper ions are cytotoxic and the resident ions in the system may bring risks in clinical applications [154]. Thus copper-free click chemistry was developed using the reactive dibenzylcyclooctyne (DBCO) with amine-containing molecules on the membrane of EVs (DBCO-EVs). Peptides comprising an azide group undergo a click-chemical reaction with the cyclooctyne on DBCO, thereby being attached to the surface of EVs [155]. The copper-free click chemistry reaction was utilized to link c(RGDyK) [58, 156], ^D^A7R [79], and CTP [157] peptides to the surface of EVs. The enrichment of modified EVs at lesion sites increased by approximately 3-fold, 1.58-fold and 2-fold.

The carbodiimide chemistry is also commonly used for engineered EVs using 1-ethyl-3-(3-dimethylaminopropyl) carbodiimide/N-hydroxysuccinimide (EDC/NHS) to covalently couple the target to amine-containing molecules on the EVs surface. AS1411 aptamer-engineered EVs using EDC/NHS displayed the highest fluorescence intensity at tumor sites after 48 h, while the fluorescence of natural EVs decreased to near-baseline levels [111, 158]. Similarly, LJM-3064 aptamer was linked to MSC derived EVs through EDC/NHS for improved treatment of multiple sclerosis.

Biotin-avidin exhibits high affinity between proteins and ligands, enabling the maintenance of interactions under harsh conditions in vivo, including low pH, high temperature and enzymatic degradation [159]. Studies have employed the phospholipids of EVs to combine with biotin to generate bio-EVs, in addition to the synthesis of biotin-modified GelMA (BioGelMA). Furthermore, affinities have been added to bio-EVs and BioGelMA to obtain complexes, which has resulted in the most potent sustained-release efficacy of the EVs and prolonged the duration of action of the EVs up to 28 days [160].

Chemical modification represents a direct alteration of the EVs membrane, a process that is relatively straightforward and requires a modest investment of time. For instance, the copper-free click chemistry method necessitates about 12–16 h [102, 156], with minimal impact on the contents in comparison to membrane fusion. Moreover, the advantages of click chemistry include high specificity and good compatibility of reaction solvents, as well as the ability to be performed in a wide range of solvents. Copper-free click chemistry is highly biocompatible, exhibiting no significant cytotoxicity [161], and is not limited by the type of parental cell of EVs. However, it should be noted that the chemical modification method utilizes specific chemical reactions such as amide bond formation and click chemistry to link the target molecules to proteins or lipids on the EVs membrane [162]. It is possible that this approach may have an impact on the natural structure and function of EVs. In addition, detailed characterization and functional verification of engineered EVs are required to ensure their safety and efficacy.

### Physical modification

The targeting of EVs may be enhanced by utilizing physical interactions, including magnetic attraction, hydrophobic interactions, or electrostatic interactions.

Magnetism targeting can be achieved by loading the EV with iron oxide nanoparticles. The loading of nanoparticles can be achieved through two main methods: extrusion and ligand-receptor interactions of transferrin. Extrusion is a frequently employed technique for the loading of iron nanoparticles. This is achieved through the utilization of a polycarbonate membrane filter with pores at nano sizes, which enables the disrupture of cellular membranes at varying sizes. Extrusion facilitates the loading of iron particles into the EV, thereby response to magnetic field and achieve targeted delivery [163, 164].

The transferrin receptor (TfR) is a membrane protein that is highly abundant in EVs. The COO^−^ groups of carboxylated arboxylated chitosan (CS) could react with the NH3^+^ groups of transferrin (Tf) to produce amido bonds in the presence of EDC and NHS sodium salt to produce Tf-SPIONs. SPION can be attached to the surface of EVs via a process known as transferrin-transferrin receptor interactions. In the presence of a magnetic field, the engineered EVs can target the lesion sites as indicated by the increased fluorescence intensity of EVs in pancreatic islets with approximately 9-fold at 10 min and 20-fold at 30 min [165].

Hydrophobicity can be employed to anchor the targeting molecules with the EV transmembrane. Since the targeting molecules assume a transmembrane α-helical configuration within the phosphatidylcholine bilayer which permits them to migrate towards the membrane surface and enter the aqueous phase, thus enabling hydrophobic insertion [166–169]. DOPE (1,2-dioleoyl-sn-glycerol-3-phosphoethanolamine) is a helper phospholipid which enhances the stability of liposomes and their capacity to merge cellular membranes [170]. It can be inserted into the surface of EV membranes through hydrophobic interactions. Furthermore, DOPE has the potential to bind to target molecules, subsequently attaching targeting molecules to the surface of EVs. The modification of MSC-derived EVs (RVG-EVs) with CNS-specific rabies virus glycoprotein (RVG) peptides, based on the aforementioned principle, resulted in improved targeting of EVs to the Alzheimer’s disease (AD) mouse cortex and hippocampus. This further enhanced the therapeutic effects of EVs on learning and memory, while significantly reducing plaque deposition and Aβ levels [171]. This principle was employed to couple IMTP to EVs, thereby demonstrating that the targeting of IMTP-EVs to the heart can also be enhanced [98, 172]. The IL4RPep-1 peptide, which targets the IL4R, was attached to the surface of EVs via hydrophobic interactions. The findings suggest that IL4RPep-1-EVs enhance the capacity to target tumors [173]. Electrostatic interaction can be employed for the targeted modification of EVs. The decrease of surface charge citroconjugated EVs (cit-EVs) was observed from the normal − 15 mV to −50 mV. In the absence of serum proteins, EVs with a high negative charge are more likely to be taken up by macrophages [174].

## Characterization of engineered EVs

The impact of distinct engineered methods on EVs was assessed in four key areas: size, morphology, stability, targeting and therapeutic efficacy. Subsequently, a statistical analysis was conducted on 30 research articles on the engineering of EVs for the treatment of neurological and cardiovascular disorders.

### Size

**Fig. 4:**
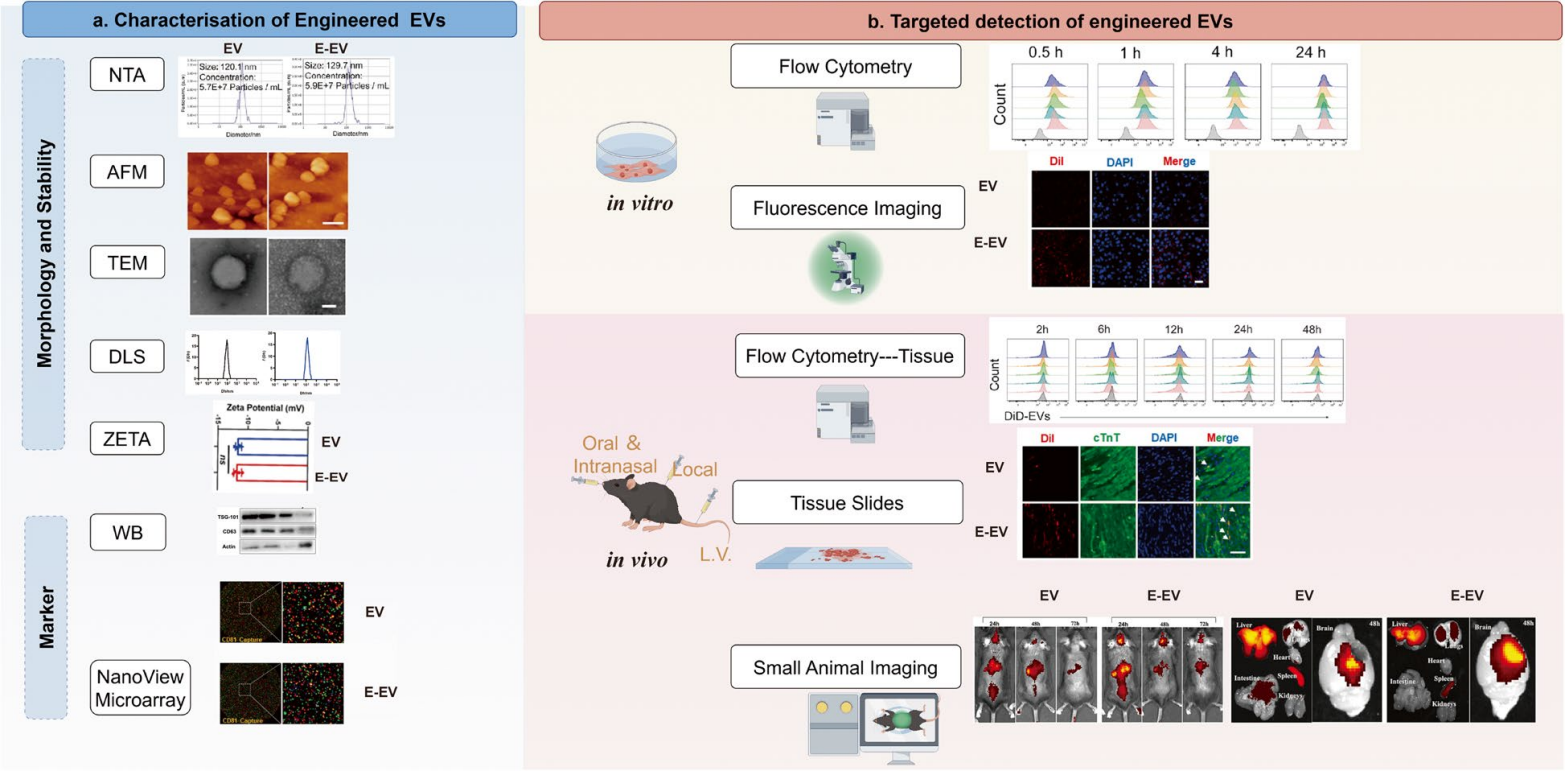
Methods for characterizing engineered EVs. Characterizations are divided into two distinct categories: EVs characterization and targeted characterization. EVs characterization includes morphological (NTA, AFM, TEM, DLS, ), stability (ZETA) and protein (WB, Nanoview microarray) characterization (**a**). Targeted characterization including in vitro (flow cytometry, fluorescence imaging) and in vivo (flow cytometry, tissue slides staining and small animal imaging) (**b**). Copyright Permission were obtained from reference [38, 107, 121]

Size of EVs were usually analyzed using nanoscale flow cytometry, atomic force microscopy (AFM), dynamic light scattering (DLS), electron microscopy, nanoparticle tracking analysis (NTA), and single-particle interferometric reflectance imaging sensor [175]. The impact of disparate modification techniques on the size of EVs was evaluated through statistical analysis, which revealed no statistically significant differences. The small variation coefficient (CV) value showed that genetically engineered EVs were more stable among publications (Table 1). However, a wide range of EV sizes was observed across publications, from 79.78 to 162.7 nm. This may be due to the variation of EV isolation, measuring principles and instruments, which requires full consideration of EV size detection (Fig. 4a). The MISEV2023 guidelines indicate that EVs are typically defined as nano- to micrometer-sized particles confined in lipid bilayers. The size range of these particles is typically between 30 and 150 nm. However, it should be noted that the size of EVs may vary depending on the cell type of origin and the biological conditions under which they are produced [176].

### Morphology

Mostly, EV morphology was imaged using transmission electron microscopy (TEM) and AFM in publications to visualize the appearance of EVs and E-EVs (Fig. 4a). TEM revealed two dimensional images in nanoscale, and the machine is more accessible than AFM. Images from an AFM represent data in 3D so that it is possible to measure the height of the nano vesicles quantitatively. Both TEM and AFM images revealed that the modification made no significant changes on the morphology of E-EVs, which were typical round cup-shaped structures.

**Table 1:** Size of Engineered EVs

Article	EV Modification	EV/nm	E-EV/nm	E-EV/EV	CV
Kang, J.Y [96]	Genetic engineering—CTP	162.70	157.30	0.97	0.03
Wang, X [104]	Genetic engineering—IMTP	134.00	135.00	1.01	
Mao, L [108]	Genetic engineering—HHP	103.00	101.00	0.98	
Li, Q [141]	Membrane fusion—platelet membrane	138.80	139.90	1.01	0.12
Hu, S [142]		79.78	114.70	1.44	
Li, Q [143]		117.50	129.20	1.10	
Zhang, N [144]	Membrane fusion—monocyte membrane	92.76	109.76	1.18	
Jiang, J [145]	Membrane fusion—Platelet membrane	91.10	111.40	1.22	
Meng, W.T [101]	Michael addition reaction—IMTP	142.00	169.00	1.19	0.16
Zhu, L.P [102]	Copper-free click chemistry reactions—IMTP	126.00	139.00	1.10	
Ruan, H [79]	Copper-free click chemistry reactions —DA7R and SDF-1	120.00	120.00	1.00	
Zhang, H [73]	Copper-free click chemistry reactions —c(RGDyK)	115.00	135.00	1.17	
Tian, T [58]	Copper click chemical modification——c(RGDyK)	92.00	131.00	1.42	
Chen, M [121]	Hydrophobic fractions—CTP	129.00	124.50	0.97	
Guo, L [177]	Carbodiimide method	135.10	137.50	1.02	
Kim, H.Y [128]	Magnetism——IONP	168.30	194.20	1.15	-

1. *E-EV *Engineered EV

### Stability

The stability characterization of engineered EVs can be evaluated through the composition of proteins and nucleic acids, the zeta potential and particle number (Fig. 4a). The expression of the EV markers, including transmembrane or lipid-binding proteins (e.g., CD9, CD63, CD81, etc.) and cytoplasmic proteins (e.g., TSG101, Alix, etc.) were characterized using WB and NanoView microarray methods. The RNA of EVs plays a pivotal role in the functionality of EVs, thus offering a promising avenue for detecting the stability of EVs. The total RNA content is usually purified and quantified using Ultraviolet–visible spectroscopy (UV-Vis) spectroscopy, while a specific mRNA or miRNA could be quantified using PCR [178]. In addition to protein and nucleic acid characterization of stability, zeta potential analysis can also detect vesicle stability [179], The zeta value is typically negative (Fig. 4a).

The stability of EVs is contingent upon a number of factors, including buffer concentration and pH value [180]. It has been demonstrated that the solution has a considerable effect on the storage of EVs, and that PBS is unsuitable for the storage. A comparison of EVs stored in PBS at + 4 °C, −20 °C and − 80 °C demonstrated a progressive decline in particle concentration, accompanied by a notable reduction in RNA levels, which decreased to approximately 50% of the initial value at + 4 °C. EVs in human albumin and trehalose to phosphate-buffered saline (PBS-HAT) was found to be 3.5 times higher than PBS, which observed by imaging flow cytometry (IFCM) [181].

### Targeting efficiency

Targeted detection was conducted in vitro and in vivo (Fig. 4b). Targeting specificity of engineered EVs were detected in vitro using flow cytometry or fluorescence imaging in examination of their uptake by targeted cells. Fluorescence imaging allows for better in situ observation. In comparison to fluorescence imaging, flow cytometry offers a more quantitative and intuitive approach to visualizing the uptake of EVs by specific cells. Co-culture of multiple kinds of cell enables a more accurate evaluation of the targeting of specific cells in comparison with that in cell culture with mono kind [182]. Targeting of engineered EV in vivo was identified through small animal imaging systems, fluorescence imaging of tissue sections, or enzymatic digestion of tissues to obtain single cell suspensions for flow cytometry fluorescence detection [107]. Small animal imaging systems allows for direct continuous detection of living subjects, but has low resolution and requires highly fluorescent dyes, and individual organs are usually separated to assess the change in fluorescence intensity of each organ for a more accurate and intuitive assessment of the targeting effect [33, 44, 125]. Tissue sections intended for fluorescence imaging detection are characterized by high resolution, but suffer from a lack of continuous observation potential and susceptible to fluorescence quenching during the preparation process. While enzymatic tissue lysis for flow cytometry can quantify fluorescence, the process of lysing cells into single cells may result in a loss of fluorescence signal and is more complicated to perform.

The labelling of EVs with fluorescent dye facilitates the tracking of EV distribution. Fluorescent dyes are available in a range of labelling types, including fluorescent, bioluminescent, radioisotope labelling and so on. Fluorescent dyes such as PKH, MemBright, DiI, DiO, DiR [183]. Lipid fluorescent dye is relatively simple and straight forward, however, it is susceptible to fluorescence quenching and photobleaching, and it lacks specific labelling [184]. Radiotracing is the most accurate method for tracking EVs, enabling the comprehensive and quantitative assessment of their biodistribution, including the analysis of pharmacokinetic processes. One study has shown that the distribution of labelling methods may vary from different label. Consequently, it is essential to exercise caution when selecting an appropriate labelling method. They assessed the impact of five distinct optical and nuclear tracers on the biodistribution of EVs in vivo, including lipid fluorescent dye DiR, the radioisotope ^111^indium-DTPA, and either fluorescent proteins fused to the EV marker CD63 (mCherry) or bioluminescent proteins (firefly and NanoLuc luciferase) to label Expi293F EVs. The study revealed that ^111^In and DiR were the most sensitive tracers for in vivo imaging, providing the most accurate quantification of vesicle biodistribution by ex vivo organ imaging and tissue lysate analysis. Furthermore, the investigation compared EVs derived from the same cellular sources and administered via the same route and dose to homozygous BALB/c mice, with the aim of specifically studying the impact of the tracers. It was observed that the genetic modification of EVs for tracing purposes, in this case through the fusion of NanoLuc to CD63, resulted in a notable alteration in their biodistribution, with a significant accumulation in the lungs [26].

**Fig. 5:**
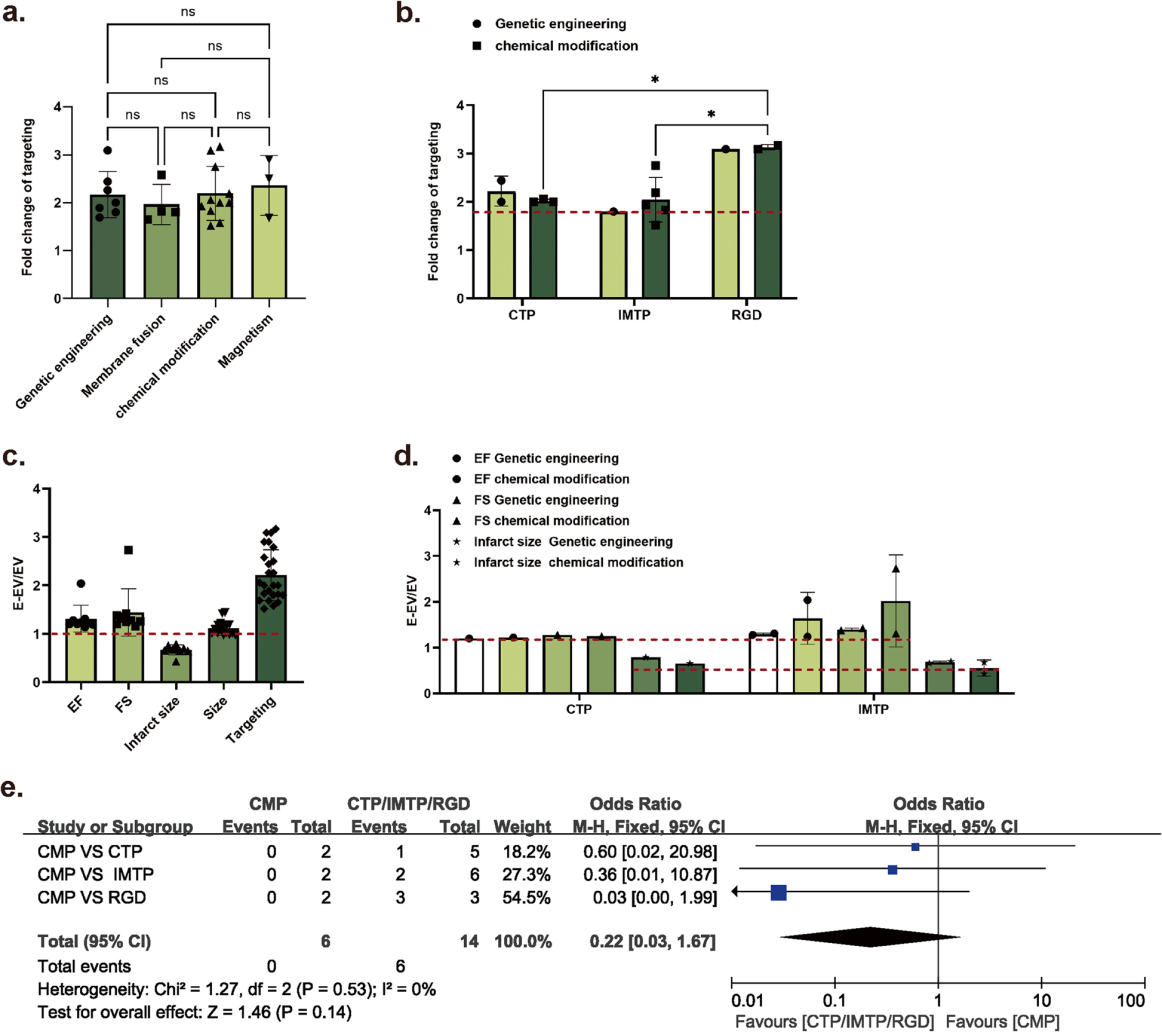
Effect of different engineering methods and molecules on targeting. **a** Effect of different engineering methods on targeting for the same targeting molecule (CTP). **b** Effect of different targeting molecules on targeting. Original data of a and b were obtained from Table 2. **c** Engineered effects on Cardiac Function (EF, FS), Infarct Size, Size of EV and Targeting. **d** Different peptide on therapeutic effect. Original data of c and d were obtained from Table 3. **a**-**d** were drawn by GraphPad Prism 9 and analyzed by One-way ANOVA. **e** Forest plot for the correlation between cardiac targeting modification molecules and targeting efficiency. Original data of e were obtained from Table 2. **e** was drawn by Review Manager 5.4 and analyzed by dichotomous variables

In order to assess the targeting efficacy of the various modification methods, the fluorescence intensity ratio was quantified before and after the engineering. One-way ANOVA was conducted to analyze the impact of the four modification techniques, namely genetic engineering, membrane fusion, chemical modification and magnetism, on targeting. The results demonstrated that these modification methods did not have a significant effect on the targeting (Fig. 5a). In addition, there was also no statistically significant difference in the impact of different engineering methods based on the modified molecules. However, the targeting molecules were found to be significantly affected by the type of modification molecules (Fig. 5b). Furthermore, additional mean values were calculated for the targeting fold change, with values over the mean representing the effective time and values below the mean defined as the ineffective time. All studies modified with this peptide was counted as total events. Statistical analyses were conducted using dichotomous variables, and the forest plot results demonstrated differences in the targeting effect of the three cardiac-targeting peptides. It was concluded that RGD, IMTP and CTP were more effective than CMP in targeting. Consequently, greater emphasis should be placed on targeting molecules (Fig. 5e). Subsequent statistical analyses were conducted to evaluate the consistency of the diverse modification techniques, as reflected by the coefficient of variation. The results demonstrated that there were notable discrepancies in the modification techniques employed by different researchers, encompassing genetic engineering, chemical modification, membrane extrusion, and magnetic (Table 2).

**Table 2:** Targeting efficiency of engineered EVs

Article	EV Modification	Measurement	E-EV/EV	Time/h	CV
Kang, J.Y [96]^a^	Genetic engineering—CTP	Small animal imaging	2.44	24	0.22
Kim, H [185]			2.00	24	
Wang, X [104]^a^	Genetic engineering—IMTP		1.80	72	
Mentkowski, K.I. [106]	Genetic engineering—CMP		1.69	24	
Mentkowski, K.I. [186]			2.26	2	
Mao, L [108]^a^	Genetic engineering—HHP		1.89	24	
Tian, T [77]^a^	Genetic engineering—RGD-4Cpeptide		3.09	24	
Li, Q [141]^a^	Membrane fusion—platelet membrane		1.80	24	0.21
Hu, S [142]^a^			1.82	72	
Li, Q [143]^a^			2.58	72	
Zhang, N [144]	Membrane fusion—monocyte membrane		1.65	2	
Zhu, L.P [102]^a^	Copper-free click chemistry reactions—IMTP	In vivo fluoresce imaging	1.83	24	0.26
Meng, W.T [101]^a^	Michael addition reaction—IMTP	Small animal imaging	2.79	24	
Mun, D [93]^a^	Copper-free click chemistry reactions—CTP		2.00	24	
Ruan, H [79]^a^	Copper-free click chemistry reactions—DA7R and SDF-1		1.58	2	
Zhang, H [73]^a^	Copper-free click chemistry reactions—c(RGDyK)		3.09	6	
Tian, T [58]^a^	Copper click chemical modification—c(RGDyK)		3.17	6	
Vandergriff, A. [98]^a^	Hydrophobic fractions—IMTP	Fluorescence imaging of Cardiac slices	1.52	24	
Lee, J.R. [100]^a^	Copper-free click chemistry reactions—IMTP	In vivo fluoresce imaging	2.26	24	
Kang, J.Y [157]^a^	Copper-free click chemistry reactions—CTP	Small animal imaging	2.00	24	
Chen, M [121]	Hydrophobic fractions—CTP		2.06	6	
Zhang, J. [18]^a^	Covalent modification—IMTP		2.90	24	
Kim, H.Y [128]^a^	Magnetism—IONP		2.90	24	0.26
Lee, J.R [164]^a^	Magnetism—IONP	In vivo imaging	1.68	24	
Liu, S [187]^a^	Magnetic -Antibody—Fe3O4@SiO2-PEG-CHO and MLC, CD63	ex vivo fluorescence imaging	2.50	0.17	

1. *E-EV *Engineered EV

2. ^a^no data in the original research article, extraction of data by Origin 2021 software

### Therapeutic efficiency

Engineered EVs, whether genetic engineering, chemical modification, membrane fusion, or physical, have the highly promising therapeutic for a range of diseases, including cancer, neurological disorders, and cardiovascular diseases. In this review, treatment efficacy was analyzed as a representative of acute myocardial infarction, with the main analyses being cardiac function and infarct size.

Cardiac function was primarily assessed in terms of left ventricular ejection fraction (EF) and left ventricular shortening fraction (FS). Due to the considerable discrepancy in the values observed across different studies, we sought to eliminate this discrepancy by comparing the values of engineered EVs(E-EVs) after treatment with those of natural EVs. The approach with EF_E−EV_/EF_EV_ >1, and FS_E−EV_/FS_EV_ >1 was assigned as an effective treatment. While in analyzing the infarct size (IS), the approach with a ratio of IS_E−EV_/IS_EV_ < 1 indicates effective treatment. The statistical results demonstrated that E-EVs exhibited remarkable efficacy in enhancing cardiac function and reducing myocardial infarct size. Additional statistical analyses of the impact of modifying molecules on the therapeutic efficacy demonstrated that the same modifying molecule did not exert a notable influence on the therapeutic efficacy (Table 3; Fig. 5c, d). Furthermore, studies have demonstrated that there was no significant toxicity on other organs by hematoxylin and eosin and enzyme-linked immunosorbent assay, which substantiates the safety of engineered EVs [185, 188].

Besides treatment of cardiovascular diseases. Engineered EVs can also be recognized as an optimal drug delivery vehicle, particularly for nucleic acids, compounds and drugs with poor water solubility, poor biocompatibility. EVs engineered by Her2, a specific tumor homing peptide, and loaded with miR-21i or 5-FU exhibited a notable effect when used in conjunction with the respective monotherapy. This was achieved by the reversal of drug resistance and the considerable enhancement of cytotoxicity in 5-FU-resistant colon cancer cells [189]. Engineered EVs with a bone-targeting peptide (AspSerSer, DSS)_6_ by means of click chemistry and then loaded them with SRT2104, a SIRT1 (silent mating-type information regulation 2 homologue 1) agonist, and the MRI contrast agent MnB NPs, utilizing electroporation. This demonstrated that the engineered EVs have excellent bone repair properties, which inhibit bone resorption and stimulate bone formation [190]. Engineered EVs have also been conducted in the delivery of compounds. In the middle cerebral artery occlusion/reperfusion model, the expression of GAP43 was increased in damaged neurons in the ischemic penumbra. Consequently, the EVs were modified with a monoclonal antibody to GAP43 (mAb GAP43), enabling the mAb GAP43-EVs to be targeted to damaged neurons with a high expression of GAP43 in the ischemic penumbra. This was subsequently employed for the targeted delivery of quercetin, a chemical found naturally in a number of foods. The findings demonstrated that the targeted delivery of mAb GAP43-EVs augmented the neuroprotective impact of quercetin [177].

Besides nucleic acids, compounds and drugs, which have proved their efficiency, new pathological findings could also be applied in developing EV-based therapy. A study employed circSCMH1 screening from the plasma of subjects diagnosed with acute ischemic stroke through the utilization of circRNA microarray. The findings indicated a notable decrease in plasma levels of circSCMH1 among stroke patients relative to the control cohort. Furthermore, this reduction was identified as exhibiting a significant correlation with the predictive capability of stroke outcomes. Based on this finding, researchers achieved targeted delivery of circSCMH1 to the brain using RVG-engineered EVs. This modified EVs were able to selectively deliver circSCMH1 to the brain, and the results suggest that engineered EVs in rodent and non-human primate models of ischemic stroke have a promising effect in promoting functional recovery after stroke. This study provides a compelling rationale for the continued exploration of EVs as a diagnostic tool in clinical settings [191].

**Table 3:** Therapeutic efficiency of engineered EVs

Article	EV Modification	Ejection Fractions (EF)			Fraction Shorting (FS)			Infarct size (IS)			
		E-EV/%	EV/%	E-EV/EV	E-EV/%	EV %	E-EV/EV	Measure	E-EV/%	EV %	E-EV/EV
Kang, J.Y. [96]	Genetic engineering—CTP	53.65	44.64	1.20	30.7	23.91	1.28	TTC	30.20	38.25	0.79
Wang, X [104]	Genetic engineering—IMTP	54.28	41.48	1.31	24.74	17.97	1.38	Masson	31.80	47.90	0.66
Wang, Y [99]		50.76	39.58	1.28	36.15	25.37	1.42	Masson	30.14	42.83	0.70
Hu, S [142]	Membrane fusion	56.22	49.41	1.14	29.54	24.59	1.20	Masson	16.29	25.08	0.65
Li, Q. [143]		55.54	46.80	1.19	29.6	25.51	1.16	Masson	16.20	21.23	0.76
Li, Q [141]		52.88	44.62	1.20	29.15	23.22	1.26	Masson	14.10	20.10	0.70
Zhu, L.P [102]	Copper-free click chemistry reactions —IMTP	47.67	38.33	1.24	32.33	24.67	1.31	Masson	8.14	18.83	0.43
Lee, J.R. [100]a		26.70	54.58	2.04	7.70	21.01	2.73	Masson	20.69	30.24	0.68
Mun, D [93]a	Copper-free click chemistry reactions —CTP	42.18	51.33	1.22	20.12	25.19	1.25	Masson	21.82	33.38	0.65

1.* E-EV *Engineered EV

2. ^a^no data in the original research article, extraction of data by Origin 2021 software

## From bench to bedside

**Fig. 6:**
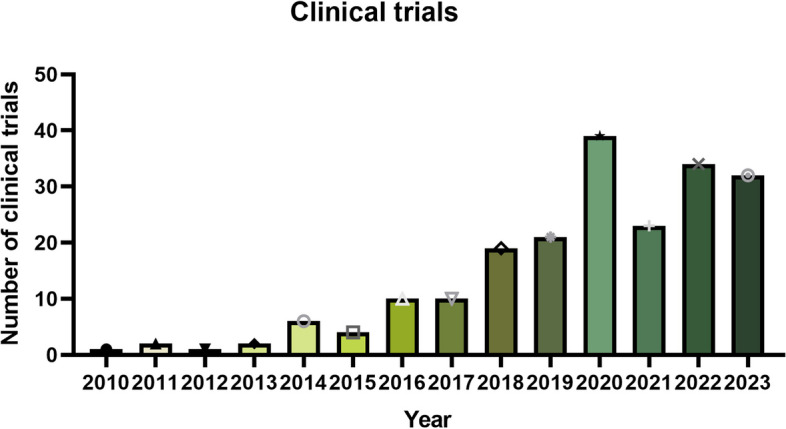
Number of clinical trials from 2010 to 2023. Original data of Fig. 6 were obtained from ClinicalTrials.gov (https://clinicaltrials.gov/), and drawn by GraphPad Prism 9

EVs have demonstrated a considerable therapy in cell and animal experiments, the number of registered clinical trials base on EV is growing. A search of the keywords “exosome” and “extracellular vesicle” on ClinicalTrials.gov (https://clinicaltrials.gov/) revealed 235 clinical studies involving EVs, as listed in supplementary Table 1. The number of clinical studies has increased significantly over the past five years, with a particular focus on the use of EVs for diagnostic, therapeutic and drug delivery purposes across a range of research areas (Fig. 6).

EVs are derived from a range of biological fluids, including urine and plasma. A current study seeks to evaluate the potential of plasma. Plasma EVs-derived miRNAs have been demonstrated to function as a biomarker for oesophageal squamous cell carcinoma [192]. A multicenter clinical study has demonstrated that EVs-based microRNAs exhibit superior performance in the diagnosis of pancreatic ductal adenocarcinoma in comparison to liquid biopsy tests that are commonly employed in clinical practice for the measurement of carbohydrate antigen 19 − 9 (CA19-9) levels [193]. The results of this study indicate that miRNAs packaged in EVs play a significant role in the pathogenesis of the disease. EVs are also an early indicator of poor prognosis in patients with ST-segment elevation myocardial infarction (NCT06070974).

In therapeutic clinical trials of EVs includes cancer, respiratory diseases, graft-versus-host disease, neurological disorders, orofacial and dermatological conditions, and aesthetic medicine. Additionally, studies have explored the potential for plant-derived EV delivery of curcumin using an oral approach to treat colon cancer (NCT01294072).

The sources of EVs in therapeutic primarily encompass both cellular and plant. The cells are predominantly of MSC origin, the MSC-EV has favorable application prospects, mainly due to MSC itself has efficacious therapeutic effects in the treatment of disease and less side effects [194]. A study on the tolerance of human adipose-derived MSC-EVs (haMSC-EVs) nebulized in all volunteers demonstrated that no serious adverse events were observed from the commencement of nebulization to day 7 post-nebulization [195]. Furthermore, a phase 2 clinical trial has demonstrated the safety and efficacy of MSC-derived EVs in the treatment of refractory perianal fistulas in patients with Crohn’s disease [196]. Studies have employed MSC-derived EVs to address atrophic acne scars. This was done in a 12-week prospective, double-blind, randomized, split-face trial of 25 patients. The findings also demonstrated that EVs are safe, in addition, it is an accessible modality for the treatment of atrophic acne scars [197]. Plant sources of EVs include ginger source, aloe source, and grape source. A completed study demonstrated the efficacy of ginger-derived EVs in the treatment of inflammatory bowel disease (IBD) and colitis-associated cancers. The study revealed that these vesicles contain high levels of lipids, low levels of proteins, approximately 125 kinds of microRNAs (miRNAs), and ginger’s bioactive constituents, including 6-gingerol and 6-gingerol [198].

Despite the existence of numerous clinical studies on EVs, the clinical trial of engineered EVs have progressed at a relatively slow pace. Only one clinical study employs focused transcranial ultrasound to enhance the delivery of growth factors and anti-inflammatory agents to localized targets, with the aim of treating depression, anxiety and dementia (NCT04202770). However, no results have been published in this ongoing trial.

Multiple modes of administration have been used in clinical trials, encompassing oral, nasal, intraperitoneal, intradermal, topical application and nebulization. Grape-derived EVs in oral administration were used in the prevention and treatment of oral mucositis associated with radiotherapy for head and neck cancer (NCT01668849). Using nasal drops, EVs derived from adipose mesenchymal stem cells have utilized in treatment of neurological disorders (NCT04388982). A recent study conducted at Tehran University of Medical Sciences in August 2024. Through intraperitoneal injection, EVs was investigated the effect of EVs in preventing early leakage in rectal cancer patients undergoing low anterior resection (NCT06536712). Furthermore, intradermal EVs is conducted for the treatment of androgenetic alopecia (NCT06239207). In addition, studies have also been conducted to investigate the impact EVs containing liquid dressings on the recovery of patients after Nd: YAG laser 532 treatment (NCT06279039). Nebulized inhalation represents a potential avenue for the treatment of lung disease. This approach has been explored in the context of lung infections caused by carbapenem-resistant gram-negative bacilli, with the use of allogeneic human adipose mesenchymal progenitor cells (haMPC) EVs. (NCT04544215).

The initial clinical trials have yielded encouraging outcomes in a multitude of ailments. Nevertheless, there are still hurdles to overcome before these findings can be translated into clinical applications. One such challenge is stability. The optimal temperature for the preservation of EVs is −80 °C, which is challenging to achieve in clinical settings and transportation [199]. Furthermore, previous studies have demonstrated that EVs are prone to aggregation in vehicle leading to blockage. At last, it is costly to produce [200]. It is necessary for further research to address issues related to preservation, transport, and monodispersity.

## Conclusions and perspectives

Engineered EVs have been demonstrated to be highly efficacy in the treatment of disease. This is achieved through the introduction of engineered modifications that facilitate precise targeting of the diseased area, while simultaneously reducing the impact on surrounding normal tissues. This approach has the potential to minimize toxicity and side effects. Furthermore, the therapeutic effect can be enhanced by increasing the concentration of EVs at the lesion site and amplifying the therapeutic effect through the loading of different therapeutic molecules. Furthermore, the half-life can be extended by enhancing the stability of the vesicles in the bloodstream, thus ensuring that a greater quantity of active ingredients reach the target site.

Furthermore, our statistical analysis of engineered EVs indicates that there is no notable impact of these on the overall size of the EVs. Additionally, the TEM results demonstrate that the morphology of the EVs remains largely unaffected by the introduction of engineered molecules.

However, engineered EVs technology needs further development. Firstly, further investigation is required to ascertain the pharmacodynamic characters of engineered EVs. In particular, the circulating metabolic pathways of EVs after entering the body require elucidation. There is a need for further development of tracking techniques in vivo. Secondly, it is recommended that further investigation be conducted into the reduced possibility of circumventing MPS and immunogenicity in order to achieve precise control of MPS while minimizing adverse effects on the innate immune system. Thirdly, it is necessary to investigate the engineering effect on structure and function of EVs. Finally, it is essential to enhance reproducibility and efficiency on modifications engineering EVs in manufacturing large scale for clinical translation.

Given the low production and high cost of EVs, which severely limit their clinical translation, it is imperative to explore EVs from different sources, such as plants, milk, bacteria, and etc. Evidence indicates that ginseng-derived EVs can effectively inhibit glioma progression and modulate tumor-associated macrophages (TAMs) [201]. Groundnut-derived EVs has been demonstrated to alleviate lipopolysaccharide-induced acute lung injury and intestinal dysbiosis [202]. Rhodiola rosea vesicles contain sRNAHJT-sRNA-m7, which directly targets the fibrotic proteins α-SMA, the ECM component fibronectin, and COL3A1, thereby improving the symptoms of pulmonary fibrosis. Furthermore, the formation of liposomes by phosphatidylcholine facilitates the entry of small RNAs into the alveoli, thereby exerting a therapeutic effect [203]. Due to their highly resistant to harsh gastrointestinal tract environments, milk-derived EVs have been used as an oral delivery vehicle of miR-31-5p to accelerates diabetic wound healing through promoting angiogenesis [204]. EVs derived from Gram-negative bacteria contain a variety of pathogen-associated molecular patterns (PAMPs), including peptidoglycan, lipopolysaccharide, and flagellin. These molecules possess a robust capacity to stimulate intrinsic immune signaling pathways, rendering them a valuable resource for the development of adjuvants and vaccine carriers. Bacterial-derived EVs were used to enhance the anti-tumor activity produced by a trained immunity-related vaccines (TIrV) [205]. Nevertheless, the intricate nature of the constituents of plant-derived or bacterial-derived EVs calls for a more comprehensive assessment of their pharmacokinetics, pharmacodynamics, safety, stability, and efficacy. Consequently, engineering various derived EVs presents a promising avenue for further advancement.

In conclusion, the therapeutic potential of engineered EVs has been significantly augmented by the introduction of targeted molecules through diverse modification techniques, thereby conferring them with extensive prospective applications in the disease management. With the ongoing advancement of research and technological developments, engineered EVs were rendered as a promising cell-free therapeutic modality.

## Supplementary Information

Supplementary Material 1.

## Data Availability

Not applicable.
